# Supplementary material for: A differential correction based shadow removal method for real-time monitoring
Source: PLoS One. 2023 Feb 7;18(2):e0276284. doi: 10.1371/journal.pone.0276284 (PMC9904483; doi:10.1371/journal.pone.0276284)
Supplement: Attachment — Submitted filename: Response_to_Reviewer.pdf [file pone.0276284.s001.pdf]

PONE-D-22-17507
Response to Reviewers' Comments

We would like to thank the editor and reviewers for their valuable comments. The actions we have taken to address their individual concerns are detailed below, where we have listed the comments in italics, followed by the responses and actions we have taken.

Journal Requirements

1. *Please ensure that your manuscript meets PLOS ONE's style requirements, including those for file naming.*

Response:

Thanks for the comment. We have revised the manuscript according to the format.

2. *Please note that PLOS ONE has specific guidelines on code sharing for submissions in which author-generated code underpins the findings in the manuscript. In these cases, all author-generated code must be made available without restrictions upon publication of the work.*

Response:

Thanks for the comment. We have shared our code at GitHub.

Action:

In the revised manuscript, we have added the code link in the last of The structure of DC-SR method subsection as follows,

" All the codes and results can be found at: https://github.com/ljx43031/DC-SR-method. "

3. *We note that you have provided funding information that is currently declared in your Funding Statement. However, funding information should not appear in the Acknowledgments section or other areas of your manuscript. We will only publish funding information present in the Funding Statement section of the online submission form.*

Response:

Thanks for the comment. We have deleted all the Funding Statement in the revised manuscrip-t.

Reviewer

In this work, a shadow removal method-based on a differential correction is proposed. The proposed method computes the differential correlation between the pixel values of the channel in RGB color space. The manuscript is interesting; however, the following comment should be addressed :

We would like to thank the reviewer very much for the valuable comments and high evaluations. We have carefully revised the manuscript, according to all comments. Please see the following for more details.

1. *Abstract : - - - - - - - - - - -*
1-Results should be included at the end of the abstract in terms of improvement ratio between the proposed and existing works .

Response:

Thanks for the comment. In order to better show the effect of our method, this paper should indeed add the improvement ratio of our method in comparison with other state-of-the-art shadow removal methods in the abstract.

Action:

In the revised manuscript, we have changed the last sentence in the abstract as follows,

"Experiment results verify that, compared with other state-of-the-art shadow removal methods, our method improves the average of object and shadow detection accuracies by at least 10% in most of the cases"

2. *Introduction Section : -*
2-The Introduction section is lengthy and need to be separated into Introduction and Related Work sections .

Response:

Thanks for the comment. We have separated the original introduction section into introduction and related work sections in the revised manuscript. Further, to better describe the proposed method, we add a few sentences to briefly analyze the existing algorithms.

Action:

In our revised manuscript, we have deleted the second paragraph and added a few sentences to briefly analyze the existing algorithms at the beginning of the original third paragraph in Introduction Section as follows,

"To satisfy the need for real-time computing in object surveillance, background difference based methods [8,9] are still the most cost-effective methods in practical application. After background difference, all the areas of objects with shadows are detected. Then, the shadows can be further removed based on one or more features which can well distinguish shadows from objects. Currently, the best feature is the RGB pixel ratio [10]. As illustrated in [10], RGB pixel ratios of shadow areas before and after shadow covering are similar, and are significantly different from the ones of object areas. Hence, the shadow areas can be found by RGB pixel ratio comparison (RGB-PRC) method."

Further, we have added a new section of Related Work as follows,

"

Related work

Currently, in the field of real-time monitoring, we still need the background difference to quickly find out the areas of objects. After that, the goal of shadow removal becomes to distinguish the shadow areas from the object areas based on two kinds of methods: the model-based and feature-based methods, respectively.

Model-based methods mainly use prior information to train corresponding models. For example, Zhang proposed a robust vehicle detection method with shadow elimination [11]. Amin Benish proposed a shadow mask extractor by using a three color attenuation model (TAM) and intensity information to segment the shadow area [12]. Saritha Murali proposed a method to remove shadows from images with uniform textures models [13]. However, those model-based methods depend on the determination of prior information, and also need a lot of training. Hence, the generalization ability of those methods are limited.

Different from model-based methods, feature-based methods mainly concentrate on distinguishing and removing the shadow by contour, brightness, color, texture and other features of pixel which are less affected by environmental factors. Hence, those methods have a wide range of application. For example, Xu obtained the stable shadow elimination results through HSV color features, by using the difference idea of image Log domain [14]. Park used shadow depth map and illumination invariance feature to remove shadows [15]. Li proposed a shadow weakening algorithm based on brightness and texture features without the prior training and manual intervention [16]. Salvador proposed a new cast shadow segmentation algorithm based on the shadow spectrum and geometric characteristics of shadows in the scene [17]. All the performance of shadow removal improved by using one or more features of images, but the computational cost is too expensive to satisfy the real time surveillance for motion object. Tang proposed a low computational cost algorithm to remove shadow according to the differences in foreground and background of the composition of pixel gray feature [18]. Chen further proposed a state-of-the-art shadow removal method: RGB pixel ratio comparison (RGB-PRC) method, based on the similar pixel change features. In this method, the shadow can be distinguished and removed directly according to the ratios of pixels between Red, Green and Blue (RGB) channels in the foreground and background [10]. Therefore, the effect of shadow removal can be greatly improved.

In this paper, we also concentrate on distinguishing and removing the shadow by pixel features. Different from the aforementioned features, the proposed pixel feature is obtained according to both the principle of shadow formation and the statistics of a large number of actual scenes. Hence, the feature proposed in this paper is more typical and has wider applicability. All of the above will be discussed in the next sections."

3. *Introduction Section : -*
3-organization of the manuscript should be included at the end of the Introduction section.

Response:

Thanks for the comment.

Action:

In our revised manuscript, we have added a paragraph of organization of this paper at the end of the Introduction Section as follows,

"The organizations of the remainder of this paper are structured as follows. In Related Work Section, the related works are described. In RGB Pixel Differential Correction Feature Section, the limitation of RGB-PRC method is illustrated, and a new RGBP-DC feature is proposed. Then, a new DC-SR method is designed based on the RGBP-DC feature in Differential Correction Based Shadow Removal Method Section. In Experiments Section, a lot of comparison experiments are performed to verify the effectiveness of the proposed DC-SR method. Finally, the conclusion and limitation of the proposed method are given in Conclusion Section."

4. *RGB pixel differential correction feature Section: -*
4-Check for grammatical errors and typos.

Response:

Thanks a lot. We have thoroughly corrected the typos and grammar errors in RGB pixel differential correction feature Section of our revised manuscript. Moreover, we have also modified some descriptions to improve the accuracy of expressions.

Action:

For example, we have made the following corrections in RGB pixel differential correction feature Section of our revised manuscript

- In the fourth sentence of the first paragraph of this Section, "Assuming the coordinate of the corresponding point of $\vec{p}$ in imaging plane is (x, y). $R(x, y)$, $G(x, y)$ and $B(x, y)$ are the pixel values of this point in R, G and B channels, respectively. $R_s(x, y)$, $G_s(x, y)$ and $B_s(x, y)$ are the ones in shadow." $\rightarrow$ "Assuming that the coordinate of the corresponding point $\vec{p}$ in imaging plane is (x, y), the pixel values of this point in R, G and B channels can be denoted as $R(x, y)$, $G(x, y)$ and $B(x, y)$, respectively, and the ones in shadow can also be denoted as $R_s(x, y)$, $G_s(x, y)$ and $B_s(x, y)$, respectively."

- In the first sentence of Analysis 1, delete "of linear relationship mentioned"

- In the first sentence of Analysis 1, "the pixel values of the corresponding point in imaging of R, G and B channels" $\rightarrow$ "the pixel values of $\vec{p}$ in R, G and B channels"

- At the last sentence of Analysis 1. "along with" $\rightarrow$ "as"

- In the first sentence of the paragraph just before Observation2, "in most image sensors" $\rightarrow$ "for most image sensors"

- In the first sentence of Analysis 2, "Assuming the nonlinear relationships between RGB pixel values and illuminance reflected from $\vec{p}$ are defined as" $\rightarrow$ "The nonlinear relationships between RGB pixel values and illuminance reflected from $\vec{p}$ are assumed to be"

- In the first sentence of Observation 3, "Assuming the differences between pixel values of the points with and without the direct light exposure in R, G and B channels are $\Delta R(x, y)$, $\Delta G(x, y)$ and $\Delta B(x, y)$, respectively." $\rightarrow$ "The differences between pixel values of point $\vec{p}$ with and without the direct light exposure in R, G and B channels are defined as $\Delta R(x, y)$, $\Delta G(x, y)$ and $\Delta B(x, y)$, respectively."

- In the sentence just before equation (15), "to obtain a stable relationship between $\Delta R(x, y)$, $\Delta G(x, y)$ and $\Delta B(x, y)$" $\rightarrow$ "and stable relationships between $\Delta R(x, y)$, $\Delta G(x, y)$ and $\Delta B(x, y)$ are obtained"

- In the last sentence of this section, "be used to distinguish and remove" $\rightarrow$ "be used to discover and remove"

- In the first sentence before (19), "Given any a small threshold T" $\rightarrow$ "Given any small threshold T"

5. *Differential correction based shadow removal method Section : - - - - - - - - - - - - - - 5-more visual example are required .*

Response:

Thanks for the valuable suggestion. More visual examples have been added in the Experiments Section to further prove the performance of the proposed methods.

Action:

In our revised manuscript, we discussed 9 examples of shadow removal cases including 6 outdoors cases and 3 indoors cases in "Fig 1 and 2", respectively.

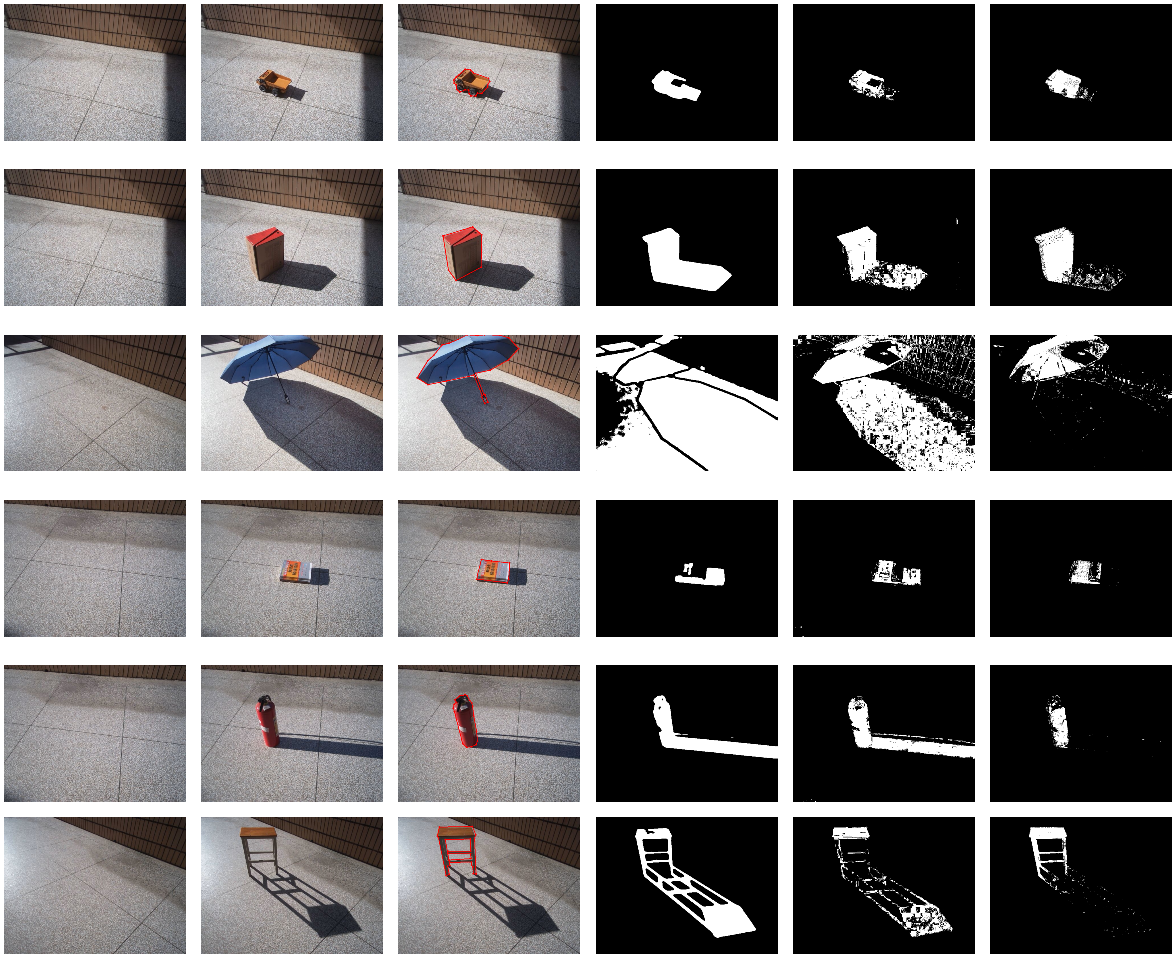

(a) Background (b) Foreground (c) Object circled in red (d) GLC (e) RGB-PRC (f) DC-SR

Figure 1: Comparison of shadow removal with GLC, RGB-PRC and DC-SR methods, respectively, in outdoor environments.

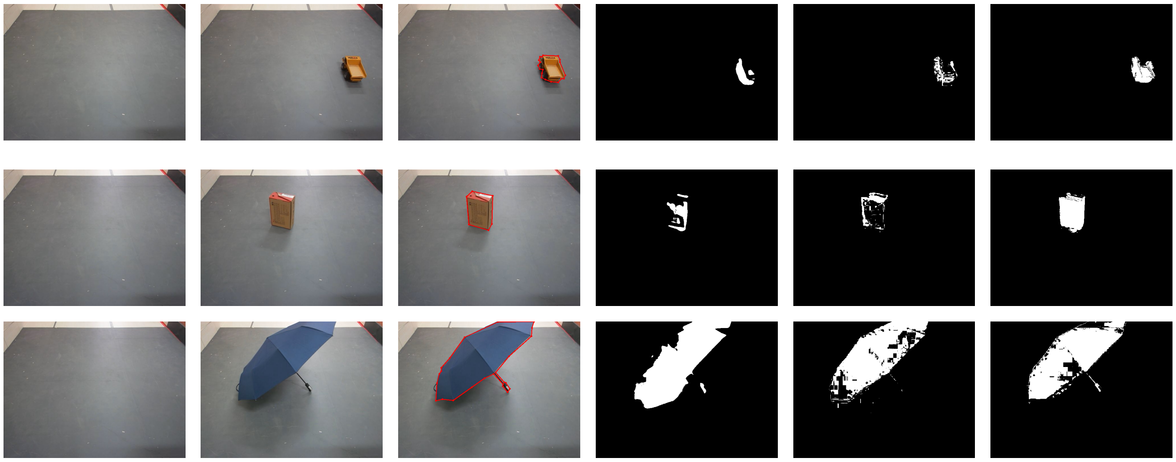

(a) Background (b) Foreground (c) Object circled in red (d) GLC (e) RGB-PRC (f) DC-SR

Figure 2: Comparison of shadow removal with GLC, RGB-PRC and DC-SR methods, respectively, in indoor environments.

6. *The structure of DC-SR method Section : - 6-Pseudo code of the method need to be included .*

Algorithm 1 DC-SR method.

Input: Foreground image $\boldsymbol{I}_f$, background image $\boldsymbol{I}_b$, the values of N_{B2R}, M_{B2R}, N_{B2G}, M_{B2G}, N_{G2R} and M_{G2R}, threshold T.
Output: The binary image of objects without shadows.

1. $\mid \Delta \boldsymbol{I}_c \mid$ is calculated as: $\mid \Delta \boldsymbol{I}_c \mid = \mid \boldsymbol{I}_b - \boldsymbol{I}_f \mid$.
2. Grayscale $\boldsymbol{I}_f$ and $\boldsymbol{I}_b$ to get $\boldsymbol{I}_{fg}$ and $\boldsymbol{I}_{bg}$.
3. Thresholding $\mid \boldsymbol{I}_{fg} - \boldsymbol{I}_{bg} \mid \rightarrow \boldsymbol{I}_m$
4. $\mid \Delta \boldsymbol{I}_c[:,:,2] \mid \boldsymbol{I}_m \rightarrow \mid \Delta \boldsymbol{R} \mid$
5. $\mid \Delta \boldsymbol{I}_c[:,:,1] \mid \boldsymbol{I}_m \rightarrow \mid \Delta \boldsymbol{G} \mid$
6. $\mid \Delta \boldsymbol{I}_c[:,:,0] \mid \boldsymbol{I}_m \rightarrow \mid \Delta \boldsymbol{B} \mid$
7. $\Delta R - [M_{B2R} + N_{B2R}\Delta B] \rightarrow \Delta\Delta R\&B$
8. $\Delta G - [M_{B2G} + N_{B2G}\Delta B] \rightarrow \Delta\Delta G\&B$
9. $\Delta R - [M_{G2R} + N_{G2R}\Delta G] \rightarrow \Delta\Delta R\&G$
10. **For:** pixel $p(x, y)$ in image:
    (a) **If:**$\Delta\Delta R\&B(x, y) < T$ and $\Delta\Delta G\&B(x, y) < T$ and $\Delta\Delta R\&G(x, y) < T$
    The value of $p(x, y)$ **is set to be 255**
    (b) **Else:**
    The value of $p(x, y)$ **is set to be 0**
    End

Response:

Thanks very much for this valuable suggestion. The pseudo code of the method has been added in the revised manuscript.

Action:

The pseudo code of the method has been added in the structure of DC-SR method Section as follow in "Algorithm 1".

Moreover, the Time complexity analysis subsection has been changed according to this algorithm as follows,

"

Time complexity analysis

As described in Algorithm 1, there are 10 steps for each shadow removal calculation. Assuming the image size is $N \times M \times 3$, step 1 contains $N \times M \times 3$ subtractions and absolute value calculations, hence the time complexity is $\boldsymbol{O}(N \times M \times 6)$. Step 2 needs to grayscale $\boldsymbol{I}_f$ and $\boldsymbol{I}_b$, which in fact averages the pixel values $\boldsymbol{I}_f$ and $\boldsymbol{I}_b$. Hence, this calculation performs two additions and one division for each pixel, and the time complexity is $\boldsymbol{O}(N \times M \times 6)$. Step 3 contains $N \times M$ subtractions and thresholding calculations, hence the time complexity is $\boldsymbol{O}(N \times M \times 2)$. Moreover, it can be easily known that the time complexity is $\boldsymbol{O}(N \times M \times 3)$ from step 4 to 6, and $\boldsymbol{O}(N \times M \times 9)$ from step 7 to 9. Step 10 is the judgements for each pixel, whose time complexity is $\boldsymbol{O}(N \times M \times 3)$. Obviously, the total time complexity of this algorithm is $\boldsymbol{O}(N \times M \times 29)$. In other words, the time complexity of this method is linearly related to the number of pixels of the video frame. "

7. *The structure of DC-SR method Section :* - - - - - - - - - - - - - - - - - - -

7-the implementation need be included as a supplementary file for review purposes .

Response:

Thanks for the valuable suggestion. The details of implementation used in this work have been directly added in the Experiments Section.

Action:

In our revised manuscript, we have added a subsection of implementation in Experiments section as follows,

“

The implementation description

In this paper, we use Hikvision 2 megapixel USB camera to take photos and use ordinary computer to run the program of the proposed method. ”

8. *Experiments Section : - - - - - - - - - - - - - - - -*
8-Experimental computation time need to be included .

Response:

Thanks for the comment.

Action:

We have added a paragraph in the Performance testing for monitoring Subsection as follows:

“Meanwhile, we test the computational time of all the aforementioned methods with the same Intel Core i7-8700 CPU at 3.2 GHz and 32 GB RAM. The results are that the proposed DC-SR method consumes 19.9ms for each removal calculation while the GLC and RGB-PRC methods consume 5.0 and 20.9 ms, respectively. Hence, the computational efficiency of the proposed DC-SR method meets the requirements of monitoring, which can be further verified that no frame drops were found in real-time monitoring experiments. Therefore, our DC-SR method can achieve efficient and accurate shadow removal in real-time monitoring.”

9. *Experiments Section : - - - - - - - - - - - - - - - -*
9-Discussion is unclear .

Response:

Thanks for the comment. To discuss the experiments more clearly, two subsections are set to analyze the static and dynamic scenes, respectively.

Action:

In our revised manuscript, we have changed the discussions as follows:

“

Analysis of shadow removal performance in static scene

Outdoor Environment: We compare the shadow removal performances of the aforementioned three methods in outdoor environment. The results are shown in Fig 1. In this figure, the first column shows the background image, the second column shows the foreground image, the third column shows the foreground image with object circled in red lines, the forth to sixth columns show the shadow removal results of the GLC, RGB-PRC and DC-SR methods,

respectively. In this bright outdoor environment, the backlit sides of the objects are very dark, which are very similar as the shadows in terms of the intensity of light reflection. Hence, those methods, such as the GLC method, which distinguish shadows relying on the intensity of light reflections will fail. This problem can be obviously seen in the forth column of Fig 1. Moreover, as mentioned in RGB Pixel Differential Correction Feature Section, the RGBP-R feature is not accurate enough to distinguish the shadow area from the object area in those bright light environments. Hence, as seen in the fifth column of Fig 1, the shadow removal performance of RGB-PRC method degrades in those environments. That is, if the object can be completely detected, the shadow cannot be perfectly removed, for example the image in the third row and fifth column. Conversely, if the shadow can be perfectly removed, the object cannot be completely detected, for example the image in the sixth row and fifth column. However, the shadow removal results of the sixth column of Fig 1 show that the proposed DC-SR method can accurately detected the object while well removing the shadow. Hence, our DC-SR method can outperforms other shadow removal methods in outdoor environment with bright light.

Indoor Environment:. As seen in Fig 2, both the performance of GLC and RGB-PRC are improved because the light intensity is much weaker than sunlight. But obviously, the proposed DC-SR method still provides the most accurate object detection results with similar shadow removal performances.

Evaluation metric: To further verify the shadow removal effect of DC-SR method, we propose the average of object and shadow detection accuracies as the evaluation metric. specifically, the essence of shadow removal is to distinguish the shadows from objects. In other words, the objects need to be correctly detected while well removing the shadows. Hence, we average the object and shadow detection accuracies to obtain a proper overall merit for shadow removal as follows:

$$\frac{1}{2}\left(\frac{N_{object}^{detected}}{N_{object}^{actual}} + \frac{N_{shadow}^{detected}}{N_{shadow}^{actual}}\right), \tag{1}$$

where $N_{object}^{detected}$ and $N_{shadow}^{detected}$ are the numbers of pixels of detected object and shadow areas, respectively. N_{object}^{actual} and N_{shadow}^{actual} are the numbers of pixels of actual object and shadow areas, respectively. To use the metric (2), actual object and shadow areas need to be known first. Hence, we manually marked the counters of the object for each case, as seen in the third columns of Fig 1 and 2, to get the actual object area. Further, we eliminate the actual object area from the difference image of foreground and background to obtain the actual shadow area. The comparison results are shown in Table 1. The cases of outdoors 1 to 6 corresponds to each row of Fig 1 and the cases of indoors 1 to 3 corresponds to each row of Fig 2. In Table 1, we can see that, in each case, the proposed DC-SR method improves the average of object and shadow detection accuracies by at least 10% except Indoors 3. But in fact, the average accuracy of proposed method is still higher that other methods in the Indoors 3 case. Hence, our DC-SR method outperforms other state-of-the-art shadow removal methods.

Performance testing for monitoring

To further verify the performance of our DC-SR method for monitoring, we test our DC-SR method under real-time monitoring. The results are shown in Fig 6 and 7. In the first rows of the two figures, the blue tracking boxes correctly frame the object area without the shadow area at any time steps. Moreover, in the second rows of the two figures, the white areas are the objects detected after shadow removal. Obviously, we can see that no shadow areas are included and the car was correctly detected.

Table 1: Accuracy Comparison.

Cases		GLC	RGB-PRC (m)	DC-SR
**Outdoors 1**	**Object detection accuracy**	0.80	0.65	0.86
	**Shadow removal accuracy**	0.45	0.82	0.80
	**Average accuracy**	0.63	0.73	0.83
**Outdoors 2**	**Object detection accuracy**	0.89	0.88	0.93
	**Shadow removal accuracy**	0.50	0.70	0.87
	**Average accuracy**	0.69	0.79	0.90
**Outdoors 3**	**Object detection accuracy**	0.64	0.85	0.74
	**Shadow removal accuracy**	0.32	0.65	0.99
	**Average accuracy**	0.48	0.75	0.86
**Outdoors 4**	**Object detection accuracy**	0.38	0.56	0.64
	**Shadow removal accuracy**	0.52	0.63	0.92
	**Average accuracy**	0.45	0.59	0.78
**Outdoors 5**	**Object detection accuracy**	0.85	0.83	0.61
	**Shadow removal accuracy**	0.47	0.48	0.99
	**Average accuracy**	0.66	0.66	0.80
**Outdoors 6**	**Object detection accuracy**	0.75	0.51	0.78
	**Shadow removal accuracy**	0.43	0.73	0.97
	**Average accuracy**	0.59	0.62	0.87
**Indoors 1**	**Object detection accuracy**	0.37	0.44	0.83
	**Shadow removal accuracy**	0.76	0.92	0.89
	**Average accuracy**	0.56	0.68	0.86
**Indoors 2**	**Object detection accuracy**	0.35	0.30	0.92
	**Shadow removal accuracy**	0.95	0.95	0.91
	**Average accuracy**	0.65	0.63	0.92
**Indoors 3**	**Object detection accuracy**	0.79	0.77	0.81
	**Shadow removal accuracy**	0.83	0.98	0.98
	**Average accuracy**	0.81	0.88	0.90

Meanwhile, we test the computational time of all the aforementioned methods with the same Intel Core i7-8700 CPU at 3.2 GHz and 32 GB RAM. The results are that the proposed DC-SR method consumes 19.9ms for each removal calculation while the GLC and RGB-PRC methods consume 5.0 and 20.9 ms, respectively. Hence, the computational efficiency of the proposed DC-SR method meets the requirements of monitoring, which can be further verified that no frame drops were found in real-time monitoring experiments. Therefore, our DC-SR method can achieve efficient and accurate shadow removal in real-time monitoring. ”

10. *Experiments Section : - - - - - - - - - - - - - - - -*
10-comparison with recent algorithm should be included.

Response:

Thanks for the comment. In fact, constrained by the computational efficiency, many neural network based new shadow removal methods are still difficult to apply to real-time monitoring. Hence, the RGB-PRC method is the state-of-the-art method. Moreover, we also added the GLC method for comparison.

Action:

In our revised manuscript, we have change the first paragraph of Experiments Section as follows,

“ In this section, our DC-SR method is evaluated in both outdoor and indoor (with sunlight lamp) environments, in comparison with RGB-PRC method and the Gray Levels Comparison (GLC) method [18]. Further, we test our DC-SR method under real-time monitoring, in order to prove its reliability and stability. For a fair comparison, the thresholds used in all the aforementioned methods are fixed. Specifically, the threshold used in our DC-SR method is set to be 8. According to [10,18], the thresholds used in RGB-PRC and GLC method are set to be 0.008 and 35, respectively. ”

Moreover, other changes in Experiments Section can bee found in the previous **Action**.

11. *Experiments Section : - - - - - - - - - - - - - - - -*
11- Evaluation metrics should be used to evaluate the performance of the proposed method.

Response:

Thanks for the valuable suggestion. To correctly evaluate the shadow removal performance, both the object and shadow detection accuracies need to be considered. Hence, this paper proposed a average of object and shadow detection accuracies metric.

Action:

In our revised manuscript, the Evaluation metric part has been added in Analysis of shadow removal performance in static scene subsection as follows,

“ **Evaluation metric:** To further verify the shadow removal effect of DC-SR method, we propose the average of object and shadow detection accuracies as the evaluation metric. specifically, the essence of shadow removal is to distinguish the shadows from objects. In other words, the objects need to be correctly detected while well removing the shadows. Hence, we average the object and shadow detection accuracies to obtain a proper overall merit for shadow removal as follows:

$$\frac{1}{2}\left(\frac{N_{object}^{detected}}{N_{object}^{actual}} + \frac{N_{shadow}^{detected}}{N_{shadow}^{actual}}\right), \tag{2}$$

where $N_{object}^{detected}$ and $N_{shadow}^{detected}$ are the numbers of pixels of detected object and shadow areas, respectively. N_{object}^{actual} and N_{shadow}^{actual} are the numbers of pixels of actual object and shadow areas, respectively. To use the metric (2), actual object and shadow areas need to be known first. Hence, we manually marked the counters of the object for each case, as seen in the third columns of Fig 1 and 2, to get the actual object area. Further, we eliminate the actual object area from the difference image of foreground and background to obtain the actual shadow area. The comparison results are shown in Table 1. The cases of outdoors 1 to 6 corresponds to each row of Fig 1 and the cases of indoors 1 to 3 corresponds to each row of Fig 2. In Table 1, we can see that, in each case, the proposed DC-SR method improves the average of object and shadow detection accuracies by at least 10% except Indoors 3. But in fact, the average accuracy of proposed method is still higher that other methods in the Indoors 3 case. Hence, our DC-SR method outperforms other state-of-the-art shadow removal methods. ”

12. *Conclusion Section : -*
12-The limitation of this work should be clearly included in the conclusion section.

Response:

Thanks for the valuable suggestion.

Action:

In our revised manuscript, the second paragraph in Conclusion Section has been added to analyze the limitation of this work as follows,

“ In fact, the performance of our DC-SR method is closely related to the parameters in (16). Although those parameters are set based on the ISTD dataset which covers the main daylight environments and represents the most common relationship between shadow and shadow-free images, in some special low-light or polarized environments, the performance of our method will still degrade. How to improve the adaptability of the method to those special environments will be an important future work. ”
